# Clinical implementation and patient‐specific quality assurance solutions for real‐time target tracking and dynamic delivery in Radixact synchrony

**DOI:** 10.1002/acm2.14545

**Published:** 2024-10-03

**Authors:** Christian D. Trujillo‐Bastidas, Michael J. Taylor, Gloria M. Díaz‐Londoño

**Affiliations:** ^1^ Clínica de Oncología Astorga, Departamento de Radioterapia/Universidad Nacional de Colombia ‐ Sede Medellín, Facultad de Ciencias Departamento de Física, Grupo de Investigación de Física Radiológica Medellín Colombia; ^2^ Accuray Inc. Deparment of Radiation Oncology Madison Wisconsin USA; ^3^ Universidad Nacional de Colombia ‐ Sede Medellín, Facultad de Ciencias, Departamento de Física Grupo de Investigación de Física Radiológica Medellín Colombia

**Keywords:** dynamic PSQA, Radixact, synchrony, tomotherapy, tracking

## Abstract

**Background:**

The installation and testing of the first Radixact with Synchrony system in Colombia marked a significant milestone in Latin America's medical landscape. There was a need to devise a robust quality assurance protocol to comprehensively evaluate both dose delivery and motion tracking accuracy. However, testing experiences under clinical conditions have not been extensively reported. Additionally, there are limited recommended measuring devices for Synchrony evaluation.

**Purpose:**

To validate and implement an alternative setup for dynamic‐PSQA while testing Synchrony's functionality under clinical scenarios, including real‐patient motion traces, and to provide guidance to new centers undergoing clinical implementation of Helical Synchrony.

**Methods:**

This approach involves using the Iba miniPhantomR with strategically placed fiducial markers for configuring Gafchromic‐films and array‐based setups. When paired with the CIRS Dynamic Platform, this enables an innovative dynamic setup with trackable features for Synchrony delivery testing. Assessment scenarios, including compensation (M1S1) and no‐motion compensation (M1S0), were evaluated using 2D‐gamma pass rate analysis with multiple clinical gamma criteria. The Synchrony‐Simulation feature was used to assess pre‐treatment performance and capture the patient's target motion pattern. Synchrony for common clinical cases with patient's motion‐traces was validated.

**Results:**

The results for M1S0 and M1S1 demonstrated consistency with previous studies evaluating Synchrony functionality. Analysis using different gamma criteria unveiled dosimetric differences and impacts across various motion ranges. The application of effective kV‐dose subtraction for array‐based methods is of upmost importance when evaluating dynamic‐PSQA with stringent gamma‐criteria. However, no significant kV‐dose impact on EBT3‐Film was detectable.

**Conclusion:**

Two implemented configurations for dynamic‐PSQA setups were validated and successfully integrated into our clinic. We addressed both the benefits and limitations of array‐based and film‐based methods. The functionality and limitations of Synchrony were evaluated using the proposed setups. The potential utility of Synchrony‐Simulation, along with the proposed patient‐case classification table, can offer valuable support for new users during the clinical implementation of Synchrony treatments.

## 1 INTRODUCTION

The Radixact system is a third‐generation helical tomotherapy system, capable of real‐time motion tracking and intrafraction motion management using the Synchrony feature. Two types of motion can be tracked: respiratory motion (RM), which is largely predictable, and quasi‐static motion (QSM), which is inherently unpredictable and includes random target excursions. While both modes can use implanted high‐density fiducials as the tracking object, allowing QSM mode to predict a target's future position and adapt the treatment delivery to target position in near real time,^1^ RM mode can use the gross tumor volume (GTV). This approach spares the patient from invasive fiducial implantation procedures, which carry risks such as pain, bleeding, infection, and pneumothorax.^2^, ^3^

In RM mode, additional continuously pulsed light‐emitting diodes (LEDs) are placed on the patient and visualized by a temporal camera. The LEDs position is correlated to the target position at any given time through kilovoltage x‐ray imaging of the patient.^4^, ^5^ The system responds quickly; however, due to the unpredictable nature of the target motion—such as prostate excursions caused by abdominal gas movement during peristalsis in QSM, or abrupt movement of a lung lesion due to patient coughing—the synchrony tracking may slightly lag behind the target.^1^

Previous works have investigated the Synchrony characteristics and performed dosimetry verification like patient‐specific quality assurance (PSQA) by evaluating both static target delivery quality assurance (DQA) and the tracking and compensating ability of the Synchrony systems for different phantom and motions traces during the DQA delivery.^6^, ^7^ For example, Chen et al. studied the dosimetric effects on DQA when combined with respiratory motion of a phantom to simulate patient motion. The team studied two dynamic motion platforms: CIRS Dynamic and ScandiDos HexaMotion Platforms. Both dynamic systems were driven with sinusoidal motion profiles. The measurement phantoms, TomoPhantom (Med Cal), ArcCheck (Sun Nuclear), and Delta4 (ScandiDos), with fiducials or high contrast inserts, were used to measure the dose distributions. The ArcCheck and Delta4 are calibrated diode array devices, while the TomoPhantom requires both a calibrated A1SL ion chamber (Standard Imaging) and Gafchromic EBT3 films (Ashland).^6^ Ferris et al. investigated the real‐time tracking and modeling for pseudo‐realistic three‐dimensional (3D) respiratory motion and clinical intensity‐modulated radiotherapy (IMRT) plans to simulate target motion. The team utilized a HexaMotion motion platform driven with sinusoidal motion profiles proposed by Lujan et al., and the dose was measured on a Delta4 for three scenarios: no phantom motion and no Synchrony (M0S0), phantom motion without Synchrony (M1S0), and phantom motion with Synchrony (M1S1).^7^, ^8^ Both Chen et al. and Ferris et al. evaluated the dose agreement with standard gamma analysis.^6^, ^7^

The Radixact system was introduced in 2017, and the Synchrony real‐time motion tracking was clinically implemented in 2019.^3^, ^4^ Although the first experiences in Latin America occurred in 2022, only implementation and verification experiences like those reported by Chen et al. and Ferris et al. were available. In 2023, publication of AAPM TG‐306 provided recommendations on QA tests and their tolerance levels.^4^ However, it encourages users to develop their own acceptance and commissioning procedures. Despite this, there have been no reports of clinical experiences evaluating real patient motion traces to date. Additionally, recommended measuring devices for Synchrony evaluations are limited. Furthermore, the use of alternative devices for dynamic QA verification has not been extensively reported. Therefore, it is necessary to provide more guidance to centers currently implementing Synchrony treatments. This includes offering additional equipment alternatives and considering clinical experiences to establish appropriate tolerance levels and criteria selection based on individual patient cases.

Considering the above, we propose an alternative and customized approach for PSQA in dynamic delivery treatments for the Radixact with Synchrony systems. This configuration involves a new setup procedure for conducting dynamic QA as part of the dosimetry and target tracking verification for each patient, similar to the standard practices used in IMRT treatments. To simulate real patient motion, we use a dynamic platform driven by actual patient motion traces extracted from the log files generated during previous Synchrony‐Simulation procedures. For dose distribution verification, we employed the Iba MatriXX‐Resolution array‐detector and Gafchromic EBT3‐Films paired with the Iba miniPhantomR. We then evaluated the dose distributions agreements using gamma analyses with regular and sensitive gamma metrics. Our findings indicate that kV‐dose from Synchrony‐radiographs can significantly impact the gamma passing rate depending on the measured device. The tests aimed to characterize and assess the performance of the Synchrony system in different common clinical scenarios, showcasing its successful implementation in a clinical setting.

## 2 MATERIALS AND METHODS

Synchrony jaw tracking on Radixact is limited to 5 cm—the maximum field size (*W*) at isocenter. Therefore, there is no Synchrony modality available for treatment plans with a 5 cm jaw setting. For RM and QSM, treatment plans modalities with the 2.5 cm jaw setting are then limited to ± 1.25 cm target longitudinal motion, while those with the 1.0 cm jaw setting are limited to ± 2.0 cm.

**FIGURE 1:**
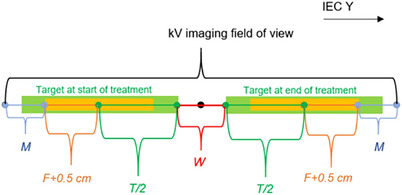
Imaging field length distribution with motion tracking during treatment. The target moves in the +IEC Y direction within the beam plane, while the imaging panel is fixed along the IEC Y axis. *M*: Target offset to fiducial limit, *F*: Fiducial spatial spread, *T/2*: Target half, *W*: Jaw field size.^9^

The green boxes in Figure 1 show the planned target's ± IEC Y positions. Initially, the target (*T*) aligns with the inferior edge of field W, and at the end, it aligns with the superior edge. For RM, the tracking centroid must be visible throughout. When using QSM, at least half the fiducials are required to remain visible. The orange box in each target marks the ± F region where fiducials are distributed around the target center in IEC Y. A ± 0.5 cm margin around each fiducial complies with detection algorithm requirements. During treatment onset and conclusion, the target may offset by ± *M* due to motion, trackable if at least half the fiducials remain within the kV imaging field of view (FOV).^9^

The multileaf collimator (MLC) is used to account for the transverse motion offset. Depending on the transverse target position, the binary MLC leaves are shifted during treatment. While the movement compensation in the longitudinal axis is continuous, in the transverse axis, it is discrete and limited by the projected size of the leaf at isocenter, which is 6.25 mm.

To predict target offset positions, bidimensional kV radiographs are acquired (up to six times per gantry rotation) simultaneously during treatment and used to generate a compensation/prediction model. The tests conducted were primarily focused on the QSM mode by delivering TomoHelical Treatment Plans with Synchrony's “Fiducial with Respiratory” mode to a detector device (chamber array and/or film) mounted on the CIRS dynamic platform.

### 2.1 Phantom preparation and image acquisition

**FIGURE 2:**
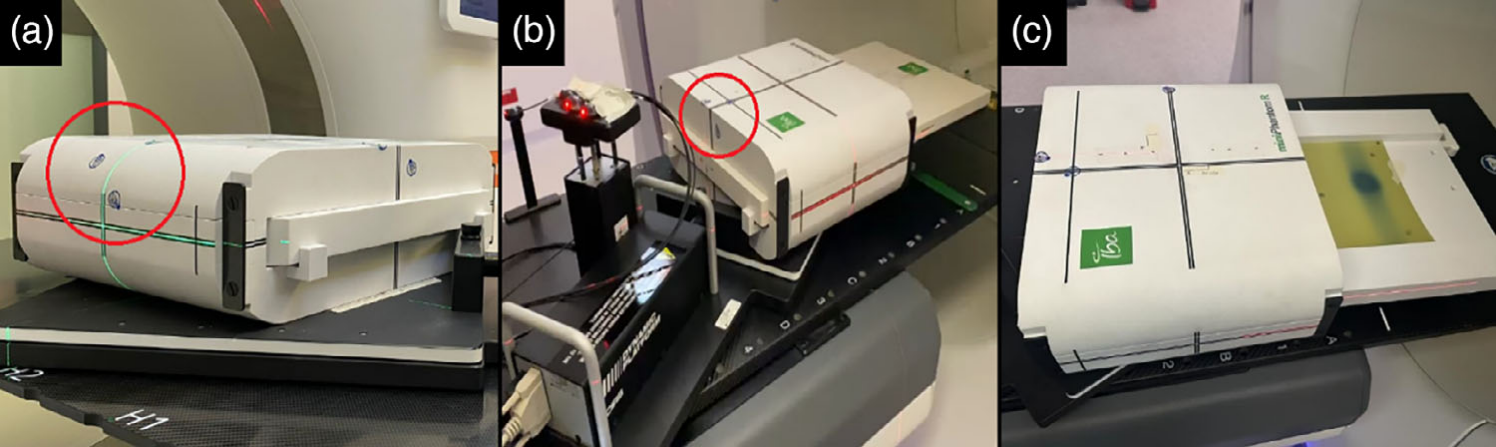
Two groups of fiducial markers were arranged on the miniPhantomR: (a) Group A for film‐based and (b) Group B for array detector based PSQA. The placement of film must correspond to the irradiation area (c).

The miniPhantomR (IBA Dosimetry GmbH) was equipped with External Fiducial Markers, as shown in Figure 2. The setup included three non‐coplanar fiducial markers—visible in kV x‐ray images—attached to the borders of the Phantom with a minimum spacing of 20 mm (to minimize fiducial overlapping) according to Accuray recommendations for fiducial‐based Synchrony treatment Plans. The miniPhantomR can hold a Film Insert or MatriXX‐Resolution detector to acquire dosimetric measurements.

CT imaging for both film‐ and array‐based setup was performed using a Siemens SOMATOM Go.Sim scanner (Siemens Healthineers, Germany). A customized radiotherapy simulation protocol was selected (120 kV, 1 mm slice thickness), with automatic exposure control on (the system selects the mA depending on the phantom size and attenuation).

### 2.2 Contouring and planning

**FIGURE 3:**
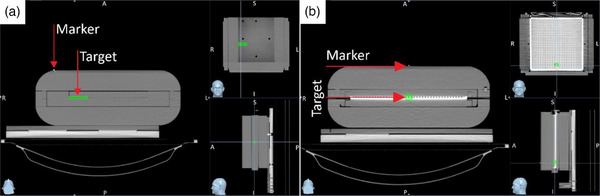
Structure contour location for (a) film‐based setup based on the fiducial markers placed on Group A and (b) array‐based setup based on the fiducial markers placed on Group B.

After CT scan acquisition, images were exported and registered as a Phantom Type on Accuracy Precision Treatment Planning System (TPS) Version 3.3.1.2. A deliverable plan was created on the Phantom, using the image set as a template for further PSQA procedures. Structures were contoured to carry out the template plan. For the film‐based setup, a structure was delineated and designated as target on the area where the film is positioned (Figure 3a). In the array‐based setup, the target structure was delimited around the chamber's location (Figure 3b). While not strictly necessary, it is advisable to position the target structure close to the fiducial markers location to ensure that the ensemble remains within the kV image FOV during the Synchrony tracking. Synchrony IMRT plans were generated for both array and film image sets using six verification angles per gantry rotation. Dose calculation was carried out with a 1.11 × 1.00 × 1.11 mm^3^ dose grid spacing using convolution‐superposition in the TPS.

As an optional step during the commissioning of Synchrony, an avoidance structure near the target can be contoured and designed as critical. This is done to generate a deliverable template plan with increased modulation, mimicking real‐patient scenario. For the film‐ and array‐based plan, a prescription dose of 50 Gy in five fractions and 70 Gy in 28 fractions was used, respectively. For this selection, we considered the possible tumor size and the device resolution.^10^ This is also based on the linearity and maximum saturation dose (up to 10 Gy) for the EBT3 Film used.^11^ Gamma analysis was performed using RIT Complete V6.11 (Radiological Imaging Technology, Inc., USA) and MyQA Patients V.2.17 (IBA Dosimetry GmbH, Germany) Software for film‐based and array‐based setup measurements, respectively. Radixact's beam quality and fluence parameters were adjusted during maintenance to achieve a gamma pass rate of 100% under 3%/1 mm criteria.

Quality control plans were established for prostate and lung cases because they represent, after breast cancer, the most common cancers globally.^12^ Besides, these cases involve a wider range of motion in real‐life scenarios and are the primary focus of the Synchrony application. Three distinct methods are employed for target tracking:

- Fiducial only: This method utilizes the tracking of small, high‐contrast fiducials, typically implanted into or near the target. Alternatively, placement in a structure rigidly attached to the target is also an option. It is designed for scenarios involving unpredictable motion, such as in prostate treatments.
- Fiducial with respiratory: Additional to fiducial tracking, this method incorporates a correlation model between the patient's respiratory cycle and the target's position. This allows for the active prediction of the target's future location during the treatment process.
- Lung with respiratory: Fiducial implantation is not required in this method. It relies on a predictive correlation model for tracking, eliminating the need for high‐contrast fiducials. However, sufficient contrast is still necessary to distinguish the target from surrounding materials, as seen in cases of tumors within the lung.

To achieve dynamic‐PSQA across a broad range of amplitudes, plans for array‐based and film‐based techniques were assigned to the “Fiducial with Respiratory” Synchrony method and saved as Derivable for later use.

### 2.3 Dynamic patient‐specific QA and delivery

A 1D motion Dynamic Platform Model 008PL (CIRS, USA), angled 30 degrees relative to the movement axis, was used to simulate motion in the XY plane. Array‐ and film‐based were positioned on the platform using additional alignment marks marked on masking tape, which were aligned with the machine alignment lasers. Because the plane of rotation of the Radixact gantry is in the X‐Z plane, the MLC shifts applied during Synchrony treatments can demonstrate effective Z motion by utilizing phantom motion in only the X direction, eliminating the need for a vertical movement of the phantom. The Y motion of the phantom will be compensated by jaw motion during Synchrony dynamic delivery.

#### 2.3.1 MatriXX‐resolution and film calibration

MatriXX‐Resolution is calibrated as suggested in the User's Guide Manual provided by the manufacturer. A helical plan was used for calibration. It can be assumed that the irradiation geometry intrinsically integrates the angular dependence of the detector.

Recommendations for the analysis and scanning of the films were carried out following the protocol of Lewis.^13^ The irradiated films were digitized 24 h after irradiation using an Epson Expression 12000 XL scanner at 150 dpi spatial resolution. The films were centered on the scanner bed in a Portrait position. TIFF images were analyzed using RIT Complete V 6.11 software. A calibration file is no longer required using the RIT's plan‐based calibration for tomotherapy patient QA feature.^14^, ^15^

#### 2.3.2 Setup validation

To validate the experimental array‐ and film‐based setup for the dynamic phantom with motion tracking, three scenarios were evaluated using the previous plans: (a) no phantom motion and no Synchrony tracking (M0S0), (b) phantom motion and no Synchrony tracking (M1S0), and (c) phantom motion with Synchrony tracking and dynamic delivery (M1S1).

1. M0S0: This represents the conventional static phantom setup and the delivery parameters for non‐Synchrony treatments. The precision of delivery was assessed employing a typical gamma criterion of 3% for dose difference and 3 mm for distance‐to‐agreement (DTA) criteria.^16^ Outcomes for all gamma analysis are deemed acceptable when the gamma criterion is met for over 95% of the data points.
2. M1S0: This test configuration was anticipated to simulate the most challenging case scenario where the phantom is in motion but there is no Synchrony tracking. During clinical implementation, harmonic waveforms were employed to induce motion in the dynamic phantom, amplitudes ranging from 3  to 18 mm with a 4 to 6‐s period range were used. Measured dose distributions were evaluated using the most common gamma criteria employed in clinical practice.^17^, ^18^ The impact of motion range, dose and target size was assessed.
3. M1S1: This configuration is representative of typical Synchrony patient treatments. It was validated that the fiducial markers adhered to the array‐based and film‐based setups were detectable by the selected Synchrony method. Measured dose distributions were compared against the calculated TPS distribution using the same motion traces and gamma criteria employed in M1S0. The impact of Synchrony tracking and delivery was assessed.

#### 2.3.3 Clinical implementation

Radixact with Synchrony has a treatment‐simulation option, which allows the user to carry out the entire Synchrony procedure without using the MV beam. This supports the therapy session by pre‐defining the imaging parameters such as kV beam angles, kV protocol, rigid body threshold, auto pause delay, etc., as well as the assessment of the accuracy of model construction and the efficiency of the delivery process. After performing the Synchrony‐simulation, a log file containing the patient's treatment data including respiratory pattern is generated and extracted.

Motion traces were extracted from log‐files generated during patient treatments. The sequence of offset position (x, y, z) during simulation was obtained for a 30 Hz sampling rate. The CIRS dynamic platform only allows one‐dimensional movement and an equivalent radiovector in the XY plane was calculated. No smoothing was applied to the resulting motion pattern to preserve the abrupt and sudden characteristics of the original patterns.

The resulting XY‐moving pattern was imported into the CIRS Motion Control software to reproduce patient breathing and movement. QA plans were created for different patient cases (prostate, lung, and pancreas) and according to the Synchrony mode, that is, fiducial, fiducial with respiratory, or lung with respiratory.

##### Patient‐specific delivery QA—array‐based

The miniPhantomR in combination with MatriXX‐Resolution detector was configurate as an array‐based dosimetry device to evaluate the effects of corrected and uncorrected target movements during helical treatment delivery. The CIRS platform has a surrogate stage where the external LED markers are placed. A reference LED is fixed to the couch to subtract the vector of couch motion from the resulting LED correlation model.

To study the independent movements on the platform along the inferior‐superior and surrogate axes (AP/LR), a constant phase shift was introduced at 50% of the cycle time. The amplitude of this shift was set equal to the independent movement observed in the XY plane.

##### Patient‐specific delivery QA—film‐based

In this complementary approach, the miniPhantomR coupled with a Film module and EBT3‐Films was used as a film‐based dosimetry device to evaluate smaller targets cases due to its better spatial resolution (up to 25 µm) compared to the array‐based system (6.25 mm). It also allows a more precise assessment of the impact for small motion ranges. The external LED markers were placed in the same way as the array‐based setup. As shown in Figure 2, the lateral placement of the fiducial markers allowed the dosimetry system to be tracked by Synchrony.

## 3 RESULTS

### 3.1 Imaging dose effect on measurements

**FIGURE 4:**
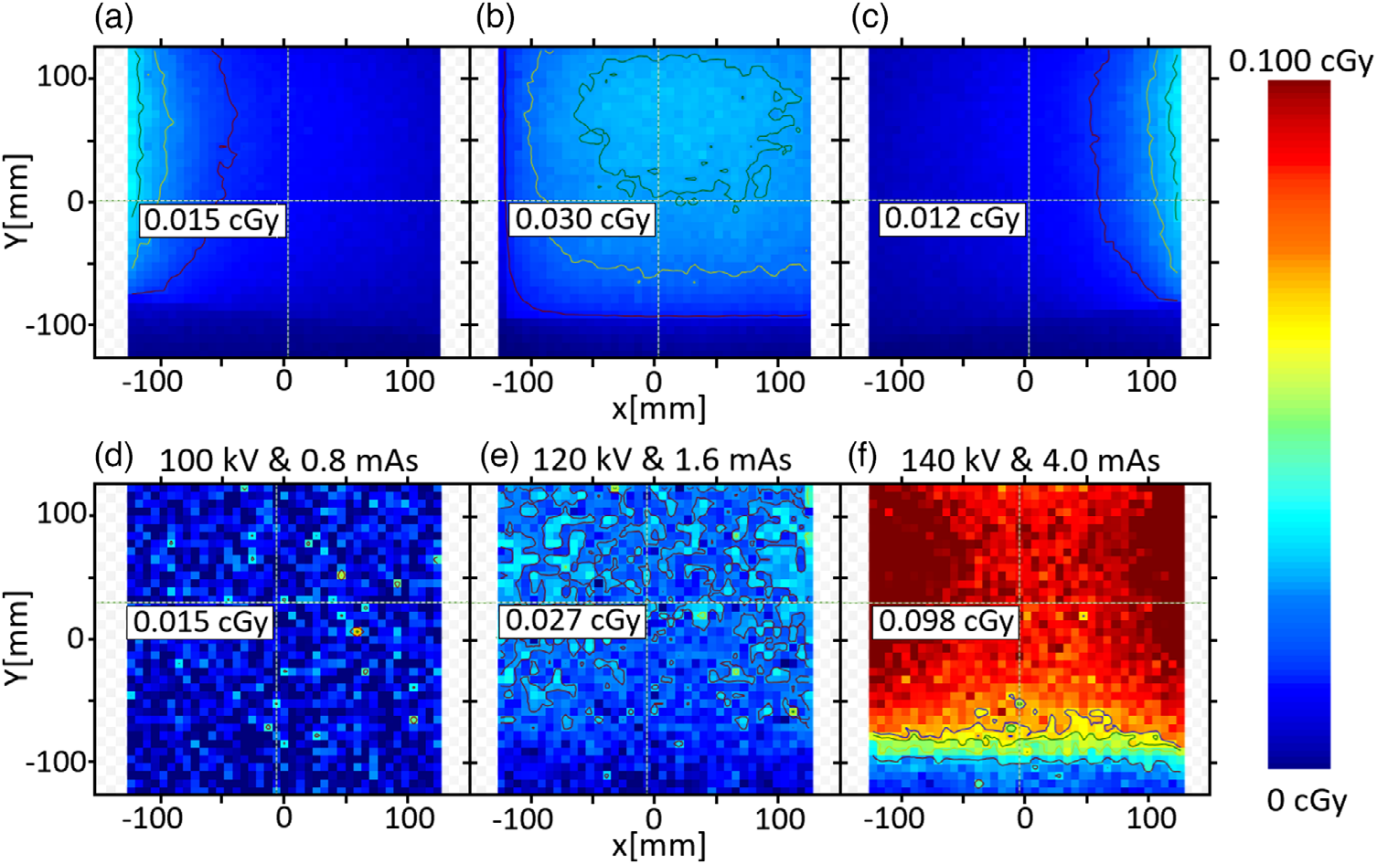
Coronal plane dose frame resulting from kV images at 60° (a), 0° (b), and 300° (c) angles for 100 kV & 0.8 mAs protocol with Array‐base setup positioned at isocenter. One gantry rotation kV‐dose contribution for six incident angles using Thorax XXS (d), Thorax L (e), and Pelvis XXL (f) kV protocols.

During array‐measurements in MyQA‐Patients software, the kV‐radiographs used by Synchrony showed an additional response compared to those from the treatment beam. Figure 4 shows a dose plane corresponding to the effect of the kV‐radiograph on the MatriXX‐Resolution detector. These frames can be subtracted from the measurement ‐one‐by‐one‐ to remove kV contamination from the total integral of measurements. However, Synchrony irradiates continuously while performing radiographic images at different preset angles, and therefore, most of the frames will correspond to MV and kV measurements. Due to the energy difference between MV and kV, it is impossible to distinguish frames that contain only MV doses from those that contain MV plus kV. Consistent with the dose‐per‐image analysis by Chen et al.,^6^ the product of the CTDI_w_ per projection with the number of images performed provides an estimate of the total kV dose. We used the same concept but estimating the cumulative kV effective dose during the PSQA measurements by integrating the contribution of images taken at different incident angles for one gantry rotation. The integrated frames were considered “uniform” (e.g., Figure 4d). The total kV‐dose contribution was exported in. csv format to calculate the average dose and used as a first approximation of the effective dose per kV gantry rotation. The resulting value was multiplied by the total number of gantry rotations used during Synchrony irradiation.

Personalized PSQA kV‐correction requires reproducing the PSQA setup and selecting the same kV‐protocol. A second approach could address the non‐uniformity of the total kV‐dose (Figure 4f) by incorporating dose profiles and considering the setup motion phase. While this might increase the complexity of the PSQA process, it may not significantly impact the gamma evaluation given the corrective scale of the kV‐dose. Nevertheless, we recommend performing dose characterization for each kV‐protocol enabled for Synchrony treatments. There can be up to six kV‐protocols (with different kV and mAs settings), each potentially resulting in varying impacts on the final deposited effective dose. While the values from these kV dose‐maps might sometimes be considered negligible compared to the total therapeutic MV dose distribution, accounting for kV contamination in measurements with array devices can lead to significantly increased gamma passing rate when dose tolerances are tightened, as shown in M1S1c of Tables 1 and 2.

**TABLE 1:** Percentage of points passing Gamma criteria for “Prostate case” during 1D motions measured with array‐based setup.

		M1S0				M1S1				M1S1c			
Gamma passing criteria (%/mm)		3/3 [%]	2/2 [%]	1/1 [%]	3/1 [%]	3/3 [%]	2/2 [%]	1/1 [%]	3/1 [%]	3/3 [%]	2/2 [%]	1/1 [%]	3/1 [%]
Motion range (mm)	0	100.0	100.0	92.5	100.0	–	–	–	–	–	–	–	–
	1	100.0	99.8	83.9	100.0	100.0	97.8	62.9	100.0	100.0	100.0	85.6	100.0
	2	100.0	97.3	68.6	100.0	100.0	99.1	67.6	99.1	100.0	100.0	84.3	100.0
	3	99.1	88.3	51.7	97.8	99.3	94.9	60.0	97.8	100	98.7	81.5	100
	5	93.2	83.9	46.5	80.7	99.3	97.6	65.3	99.1	100	99.3	84.2	100
	7	94.4	79.6	44.5	74.4	100.0	96.0	65.4	100.0	100	100	81.1	100
	10	83.8	54.4	23.4	59.3	99.6	98.4	73.0	98.2	100.0	98.9	80.2	99.3

A 5% threshold dose was applied. M1S1c represents the dose correction due to the array detectors over‐response during kV imaging following the procedure described previously.

**TABLE 2:** Percentage of points passing gamma criteria for “Lung case” during 1D motions measured with array‐based setup.

		M1S0				M1S1				M1S1c			
Gamma passing criteria (%/mm)		3/3 [%]	2/2 [%]	1/1 [%]	3/1 [%]	3/3 [%]	2/2 [%]	1/1 [%]	3/1 [%]	3/3 [%]	2/2 [%]	1/1 [%]	3/1 [%]
Motion range (mm)	0	100.0	100.0	100.0	100.0	–	–	–	–	–	–	–	–
	1	100.0	98.3	71.7	100	100.0	97.2	56.1	99.6	100.0	100.0	96.5	100.0
	3	100.0	98.3	77.3	100.0	100.0	98.8	62.0	100.0	100.0	100.0	97.0	100.0
	6	98.0	90.8	73.1	97.9	99.6	95.4	55.0	97.9	100.0	99.6	93.1	100.0
	9	92.8	81.0	65.8	81.4	98.8	96.3	58.9	97.5	99.6	98.7	92.2	99.1
	12	84.9	70.6	56.6	71.3	100.0	93.3	52.9	95.4	100.0	99.1	89.0	99.6
	15	61.5	61.1	38.9	61.5	98.8	93.4	62.1	95.9	99.1	98.7	89.2	97.8
	18	60.9	37.9	16.9	47.6	99.2	93.9	52.9	97.1	99.6	98.7	89.9	97.4

A 5% threshold dose was applied.

On the other hand, film kV‐dose corrections can be considered negligible. Gafchromic EBT3‐Film specifications^19^ report an optimum dose range from 20 to 1000 cGy, which is significantly higher than the effective kV dose from a Synchrony radiograph, as indicated by the overestimated values in Figure 4a–c. Additionally, EBT3‐films exhibit significant energy dependence for kV‐range from 100 to 150 kVp, with an underestimation of 15%–17% for 100 cGy of absorbed dose, according to Villarreal‐Barajas et al.^20^ Although no information for low doses (< 25 cGy) with kV‐energies is reported,^21^, ^22^, ^23^ underresponse and film inability to measure accurately within the optimum dose range for kV‐dose are expected.

### 3.2 Prostate mock case

Proposed PSQA array‐ and film‐based setups were tracked using Synchrony “Fiducial with Respiratory” method, allowing the dosimetric ensemble to be useful for measuring the dynamic effects of moving targets and assessing accuracy of Synchrony.

A mock Prostate plan for a moderate hypofractionation case (2.5 Gy/Fx) was generated using a 2.5 cm jaw setting. Gantry period and beam on time was 12.1 and 140 s, respectively. Although prostates exhibit rather random and abrupt movements, a sinusoidal motion pattern was employed solely to validate the system's tracking performance and capability. Additionally, it was used to assess the potential impact of different amplitudes on dose distribution. M1S1 for motion ranges (*half‐amplitude*) above 10 mm are not reported due to maximum jaw ranges realized for the 2.5 cm jaw plan. The agreement using gamma‐criteria is shown in Table 1.

**FIGURE 5:**
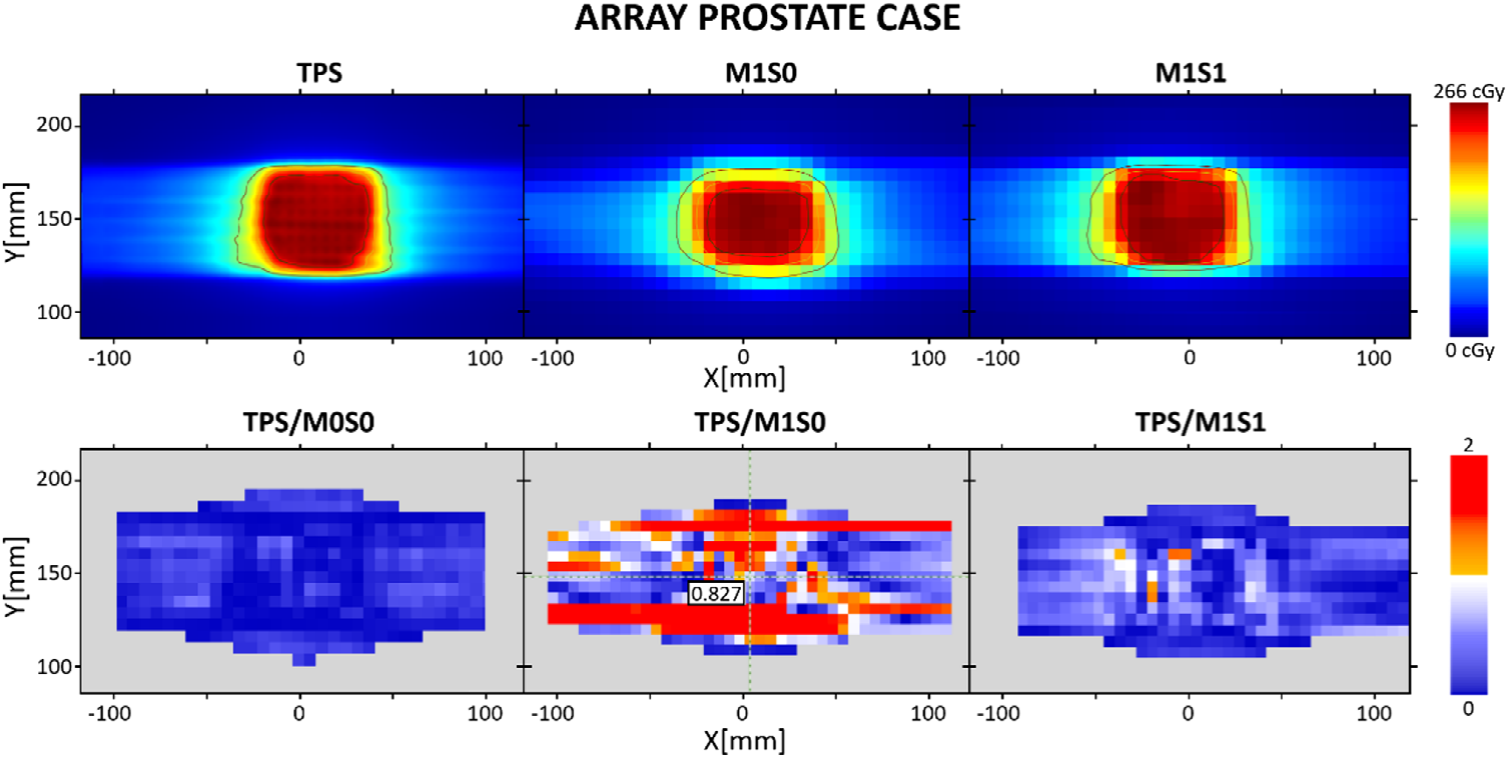
Results for prostate case with 10 mm peak‐to‐peak motion range using sinusoidal motion trace. Top row: Coronal plane dose distribution. Bottom row: gamma index maps for 3%/3 mm criteria.

For M1S0, it is evident that as the motion range increases, the gamma passing rate decreases, as expected due to the distortion effect on the dose distribution. In our case, given the type of movement of the platform, the distortion occurs symmetrically on the axis of movement, as shown in Figure 5.

The total kV‐dose contribution for Pelvis XXL protocol during the prostate case setup was 2.6 cGy. This value was subtracted from the measured dose map. As shown in Table 1, this correction generates an increase of the gamma passing points evaluated at 3%/3 mm criteria. For tighter criteria, with some exceptions, this correction does not increase the number of passing gamma points. As shown in Figure 4f, the incident angled kV images do not cause a uniform over‐response in the entire detector array, which implies that the points in the array that were least affected by the kV image dose are going to have a post‐correction with an under‐estimated value.

**FIGURE 6:**
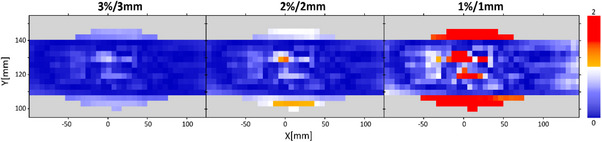
Gamma index maps for 3 mm amplitude sinusoidal motion when comparing Reference TPS versus M1S1 treatment.

As shown in Figure 6, the gamma index maps show indicate that the upper and lower regions (cranial and caudal in the XY plane), which correspond to the adjacent radiation areas outside the main radiation field where only secondary radiation was detected, exhibit less over‐response during measurement than the chambers within the radiation field, which received primary doses of MV and kV. This suggests that kV‐dose correction should be performed using dose‐maps subtractions rather than a single average value.

The observed outcomes carry substantial clinical relevance, especially in evaluating expansion margins from GTV to planning target volume (PTV). Positive results from PSQA evaluations under more stringent criteria indicate the system's ability to accurately track the treatment target. This capability ensures precise irradiation of the intended volume, suggesting that the use of extensive expansion margins may not be as imperative. Consequently, conducting a dynamic‐PSQA provides an additional tool for accurately selecting expansion margins.

### 3.3 Lung mock case

For evaluation purposes, the lung plan originally created on the film‐based setup was also performed on the array‐based setup in QA mode. An equivalent stereotactic ablative radiation therapy (SABR) plan (10 Gy/Fx) was generated using a 1.0 cm field width. The gantry period and beam‐on time was 12.5 and 507.6 s, respectively. The agreement using gamma‐ criteria is shown in Table 2.

**FIGURE 7:**
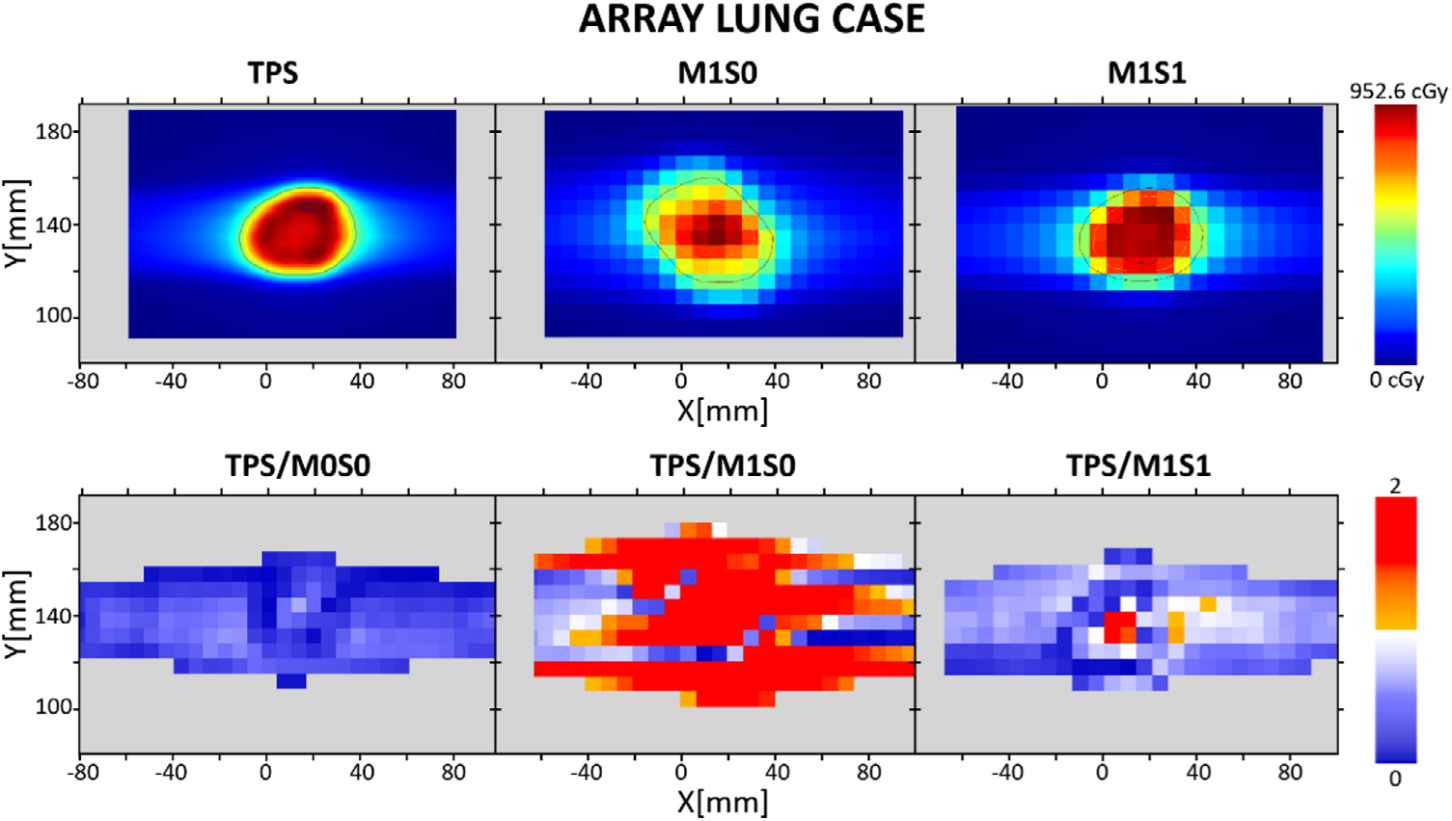
Array‐based results for lung case with 18 mm peak‐to‐peak motion range using sinusoidal motion trace. Top row: Coronal plane dose distribution. Bottom row: gamma index maps for 3%/3 mm criteria.

Similar to the prostate case, the gamma passing rate for M1S0 decreases as the motion range increases. A target XY‐size was defined as the root of the quadrature sum for the largest value in X and Y. However, compared to the previous case where the relation between the maximum motion range and the target size was up to ∼14.5%, in this case, it is up to ∼25.6%. Figure 7 illustrates how the isodose distortion and the gamma passing map values lose correlation with the reference dose distribution. The average total kV‐dose contribution for Thorax L protocol was 11.6 cGy. This value was subtracted from the measured dose map and reported in the M1S1c column in Table 2.

The gamma analysis for array‐based QA further evidences the intrinsic error due to the detector resolution in comparison to the target size and the used motion ranges during dynamic measurements. This causes a partial volume effect in the chamber‐array measurement. Despite this, as shown in the M1S1 column of Table 2, the operation and performance of Synchrony can still be effectively evaluated. Moreover, better results are achieved when kV dose correction is applied.

**FIGURE 8:**
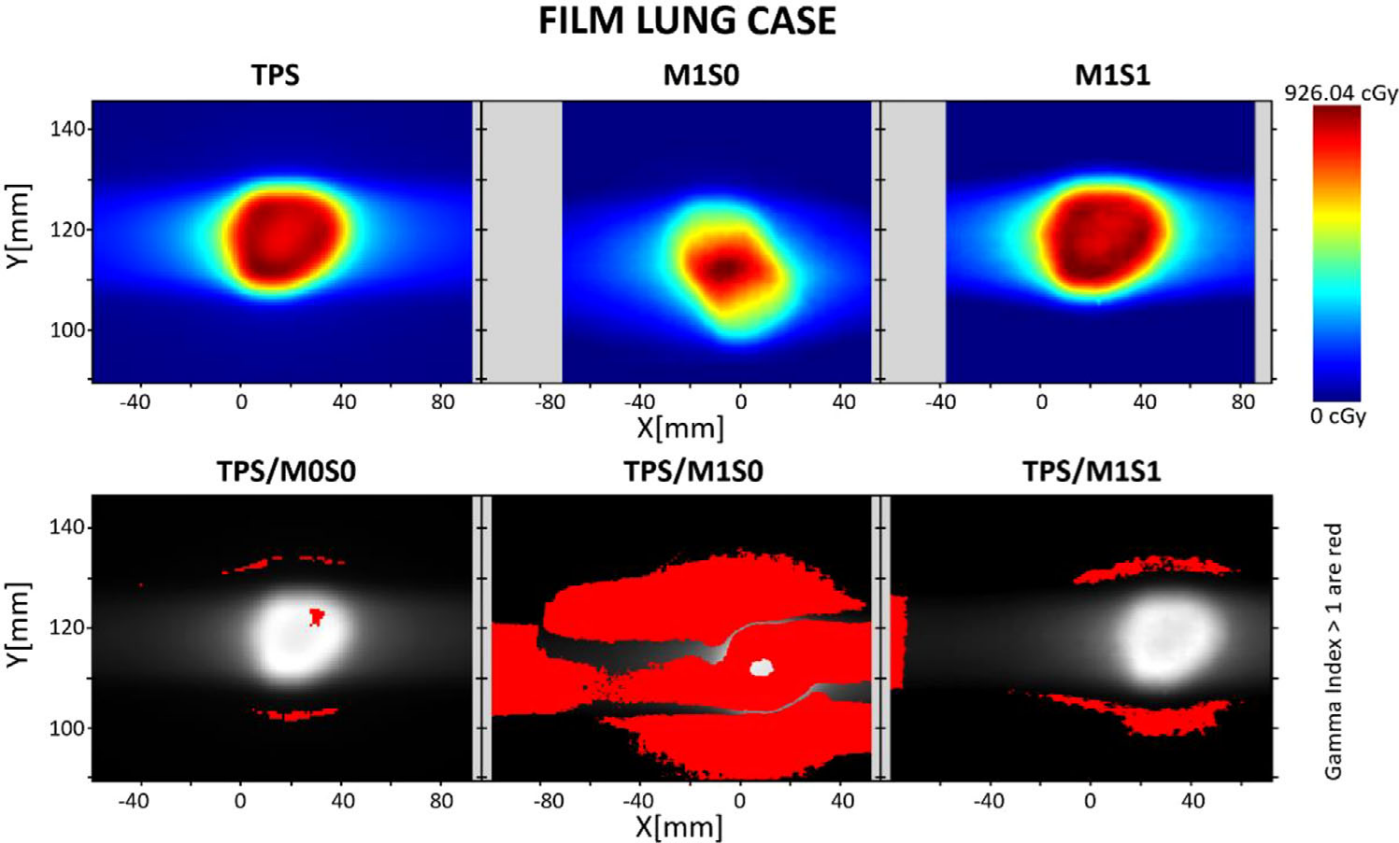
Film‐based results for lung case with 18 mm peak‐to‐peak motion range using sinusoidal motion trace. The top part displays the coronal plane dose distribution, while the bottom row shows gamma index maps for 3%/3 mm criteria.

Figure 8 demonstrates a better resolution of the distortion effect compared to Figure 7 using the array‐based setup. The agreement using gamma passing criteria is presented in Table 3. No M1S1c is proposed for the film‐based setup.

**TABLE 3:** Percentage of points passing gamma criteria for “Lung case” during 1D motions measured with film‐based setup.

		M1S0				M1S1			
Gamma passing criteria (%/mm)		3/3 [%]	2/2 [%]	1/1 [%]	3/1 [%]	3/3 [%]	2/2 [%]	1/1 [%]	3/1 [%]
Motion range (mm)	0	97.7	87.9	35.7	86.0	–	–	–	–
	1	94.2	76.9	27.2	72.1	94.9	84.0	37.9	72.4
	3	94.9	72.5	19.7	56.5	96.3	81.3	40.1	75.6
	6	89.7	63.3	19.8	58.3	95.5	83.3	36.5	73.7
	12	42.2	23.8	6.8	19.8	99.5	83.4	30.9	92.9
	18	39.4	24.8	8.9	27.9	95.7	77.8	27.4	71.6

A 5% threshold dose was applied.

For cases with high distortion (when Motion range > 6 mm), RIT's plan‐based calibration for tomotherapy patient QA could not be applied. In these instances, a manual registration was performed to achieve the best concordance between the dose distributions. Additionally, a finely tuned registration was applied.

### 3.4 Synchrony tracking limitations

#### 3.4.1 Upper limit of motion

As mentioned previously, there is a ± 20 mm superior/inferior jaw tracking motion limit for a 10 mm jaw plan. However, in our Radixact system, amplitudes greater than ± 19 mm cause continuous interruptions, leading to repeated rebuilding of the tracking model until the system can adapt. This can incur a significant increase in the number of kV images required, potentially doubling the number compared to when the target is within the tracking range. To mitigate the potential impact on patient treatment time, which can include exhaustion, and more erratic and agitated breathing pattern, our clinic has established an 18 mm peak‐to‐peak motion threshold for target range.

This motion range problem could be partially addressed by increasing the tolerance of the “Target Outside Jaw Range Threshold” parameter, allowing the treatment to continue even when the target is outside the Synchrony maximum range. However, this approach introduces an undesirable level of uncertainty in the irradiation of the target´s extremities. For those clinical cases where the target moves more than 18 mm, increasing the margin of the PTV could be advisable. This increase in volume of irradiation may compensate for the reduced dose at the extremities when the target movement exceeds tolerance.

In the case of plans with 2.5 cm jaw setting, nominal jaw tracking could not be achieved. For amplitudes greater than 10 mm, we encountered similar limitations as mentioned for 1.0 cm jaw setting.

#### 3.4.2 Lower limit of motion

**FIGURE 9:**
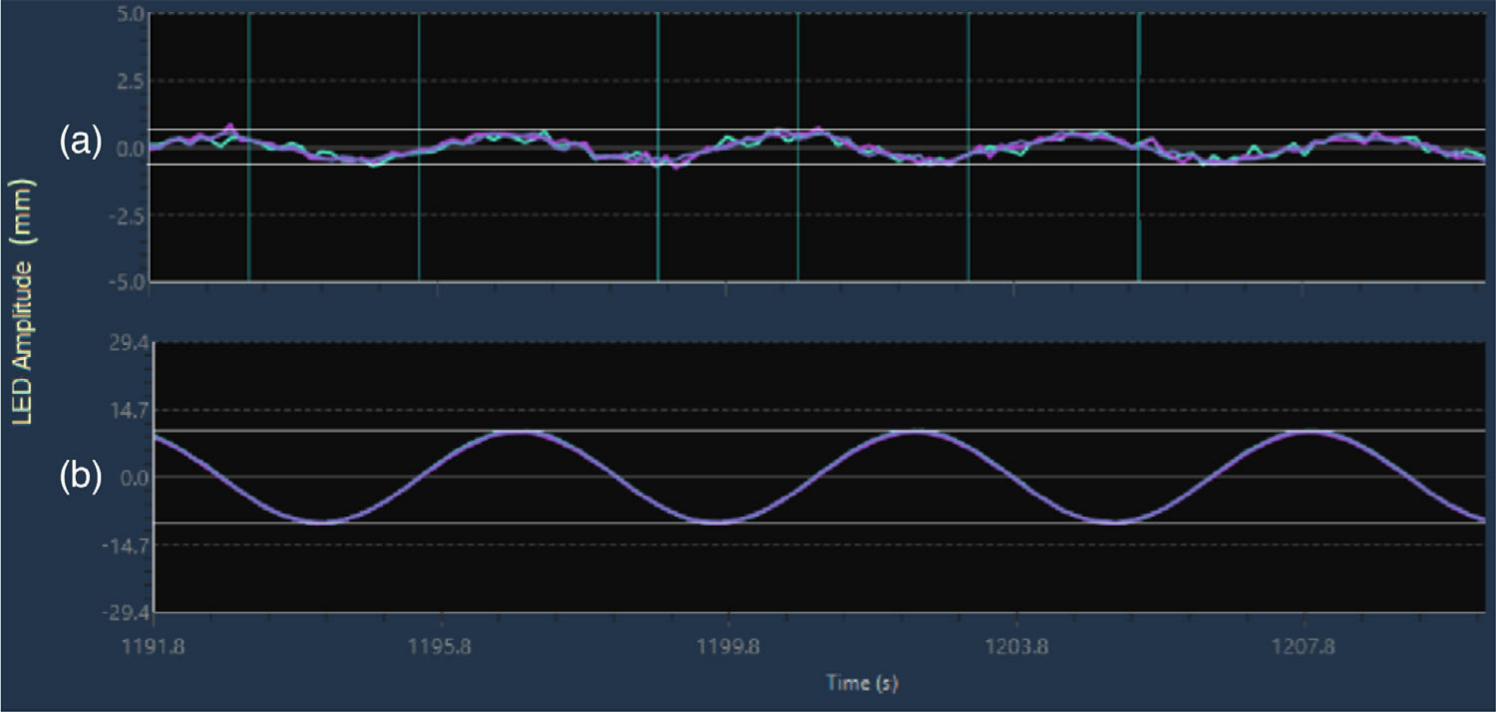
LED motion range for: (a) 0.7 mm and (b) 20 mm motion ranges.

The minimum motion range traceable for the Synchrony system at Clínica de Oncología Astorga was 0.7 mm. Below this threshold, the LED graphs (Figure 9) appear to be within the signal noise level, and warnings such as “Low LED Motion for Model” or “Synchronization could not be computed. Rebuild Model” prevent the system to create a model. However, for target motion and LED amplitudes under 1 mm, there is no apparent distortion of the dose distributions. This is reflected in the gamma passing rate, which shows practically equal values for M1S0 and M1S1. This consistency occurs because the DTA‐criteria is of the same order or even higher than the target motion amplitude.

However, this result may impact the selection and eligibility of patients for synchrony. For targets located in quasi‐static areas with reduced movement, and where a fiducial implantation is required for tracking, the benefits of the technique might not be substantial enough to justify the invasive process and additional kV dose for the patient.

It should be considered that this result was obtained for a target whose size (50 cc Prostate) and dose (2.5 Gy/Fx) could mitigate the impact of this motion range compared to a smaller target, such as a brain metastasis (as low as 1 cc) and a single fraction dose of 22 Gy; clinically, there may be cases where irradiation precision is significantly impacted by submillimeter corrections, especially when organs‐at‐risk (OAR) are adjacent to or near the target.

#### 3.4.3 PSQA implementation

**FIGURE 10:**
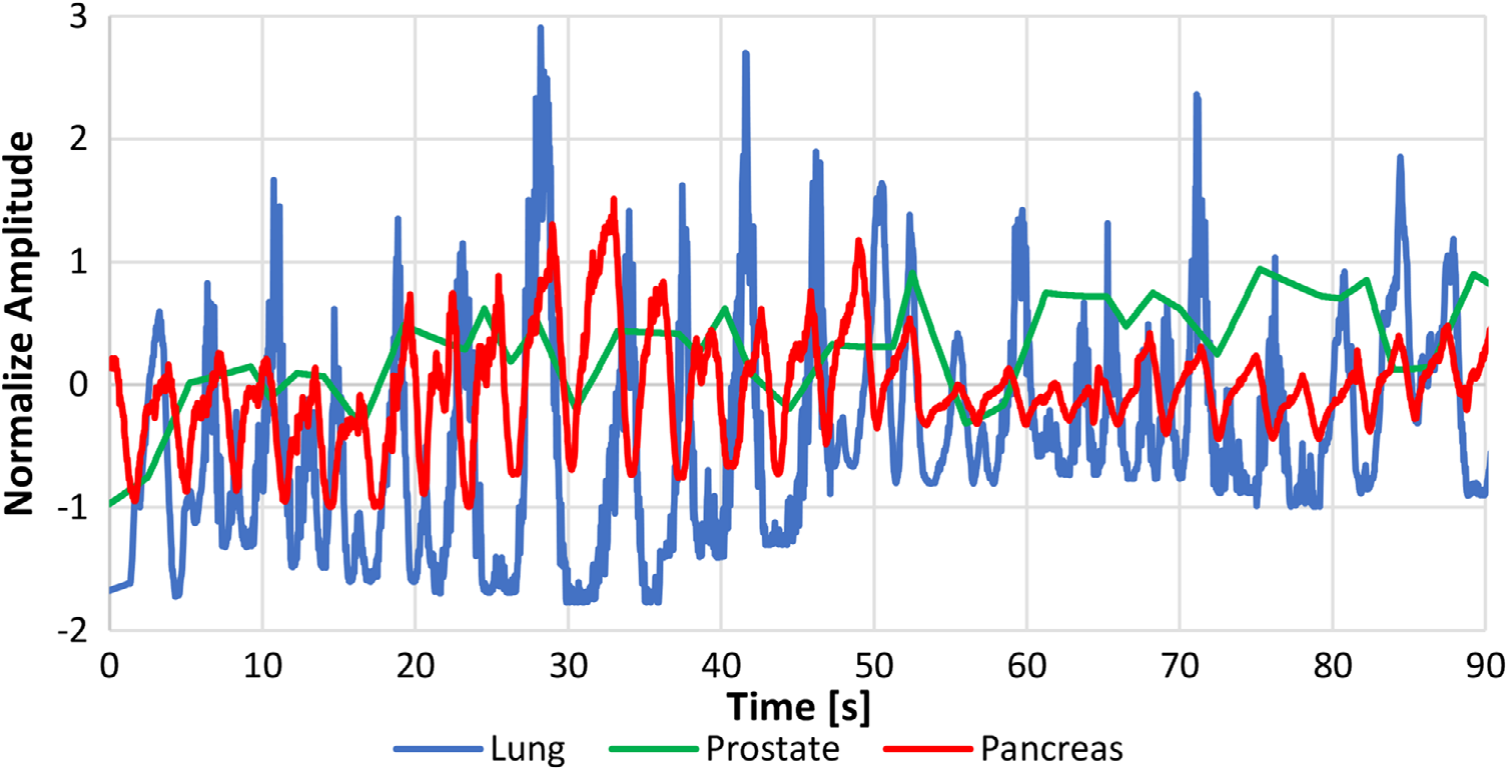
Real patient motion traces for different synchrony modalities. “Lung,” “Pancreas,” and “Prostate” correspond to “Lung with respiratory,” “Fiducial with Respiratory,” and “Fiducial only” Synchrony modalities, respectively.

Before treating each patient, a synchrony simulation was conducted to extract patient specific motion traces and to define the final planning optimization parameters, including field width and pitch. After treatment plan approval, PSQA was generated using the plans previously created as Templates. A routine PSQA setup was performed, incorporating the CIRS dynamic platform. For each case listed in Table 4, the corresponding motion patterns were imported. Figure 10 illustrates the motion pattern for some of the evaluated cases.

**TABLE 4:** Percentage of points passing gamma criteria for pre‐treatment PSQA using patients motion pattern.

	Motion amplitude (mm)	Target XY‐size (mm)	Dose per fraction (Gy/Fx)	Criteria^a^	Gamma passing rate (M1S1c) (%)
Patient 1 ‐ Lung	9.37	39.0	10.0	3%/1 mm	100.0
Patient 2 ‐ Prostate	0.83	69.0	2.5	2%/2 mm	100.0
Patient 3 ‐ Pancreas	3.84	40.3	8.0	2%/2 mm	96.9

The motion amplitude denotes the radiovector in the XY plane, calculated by taking the maximum amplitudes of both X and Y axes during respiratory motion.

^a^ Gamma‐passing rate must pass 95% and action level 90%.

It is noticeable that in cases where the respiratory pattern is included, a harmonic behavior is observed (lung and pancreas), in contrast to the prostate case, where a random movement is evident. Table 4 summarizes the results and characteristics of the PSQA measured. All cases were assessed using the array‐based setup.

The number of kV radiographs required during Synchrony treatment can vary widely, depending on factors such as treatment beam‐on time, the preset number of kV radiographs per gantry rotation, the patient's respiratory patterns, physiology, and health condition. However, the user's ability to set optimal tracking‐parameters plays a pivotal role. Standardized thresholds values for Synchrony parameters are not reported, as they are specific to each patient. During our learning curve, some cases exhibited tracking difficulties, leading to situations where up to three times the number of kV radiographs used during PSQA were required to deliver the treatment.

Table 5 outlines our proposed patient case classification (PCC) system, based on the dosimetric findings from M1S1/M1S1c deliveries as function of gamma‐criteria and target's motion range.

**TABLE 5:** PCC for motion tracking and compensation requirement.

Motion range (mm)	Gamma passing criteria	Motion tracking and compensation	Possible/Comparable clinical outcome	kV dose correction (*if applied*)
≤ 3	Any^a^	No	Conventional IMRT	Negligible^b^
≤ 3	3%/3 mm 3%/1 mm	Yes	Conventional IMRT SABR	Recommended
3–6	2%/2 mm	Recommended	SABR	Recommended
≤ 6	3%/3 mm 3%/2 mm	Depends on tumor size and dose	Conventional IMRT SABR	It may be omitted^c^
> 6	As tight as possible^d^	Required	SABR	Recommended

^a^ Relaxed or tight tolerance must pass 95% and action level 90%.

^b^ Considering set up positioning images only.

^c^ According to TG‐306 Gamma Criteria.

^d^ Depends on own clinical protocols.

## 4 DISCUSSION

### 4.1 Setup validation

Machine quality controls, as recommended in AAPM protocols,^4^, ^24^, ^25^ were performed during regular system calibration using ion chamber point measurements. High gamma passing rates for M0S0 cases were found: 100% for prostate and lung with the array‐based measurement technique even under tight criteria of 2%/2 mm and 97.7% for the film‐based with 3%/3 mm criteria.

The M1S0 case dose showed a decreasing trend in accuracy as the target motion range increased for all cases. Similar results were reported by Goddard et al.^26^ using ArcCheck and Delta4 Phantoms. This is not at all surprising: as the motion increases, the measured dose accuracy will decrease if the motion is not accounted for with dynamic delivery. The film measurements also exhibited this trend for 3%/3 mm criteria. However, no correlation was found with tighter‐gamma criteria, possibly due to the handling and uncertainty in the film registration. Additionally, the effects of dynamic‐film measurements, such as additional dose‐smearing associated with film delayed radiation‐response, need further investigation. As shown in Figures 8 and 9, larger motion ranges resulted in more distorted dose distribution, which is reflected in the lower gamma pass rates.

The M1S1 case showed an improvement in gamma passing rate; The dose distribution maps in M1S1 approached the M0S0 case, with gamma passing rate > 95% for all cases. While analyses of different target sizes and dose per fraction exhibit similar behaviors, both cases indicate a significant impact on treatment delivery when motion tracking and compensation are employed. For the M1S1c case, improved gamma results were observed across all evaluated gamma criteria, particularly for those with reduced dose criteria tolerance, even when these were below the passing threshold. This highlights that, although the dose per image may be considered negligible relative to the delivered dose, it can lead to a significant increase (up to 22% and 40% for the prostate and lung cases with 1%/1 mm criteria, respectively) in the gamma passing rate, for an entire treatment fraction.

When comparing M1S0 and M1S1 up to 3 mm motion ranges for the array‐based setup, the gamma index in both scenarios does not show significant differences for 3%/3 mm criteria (under < 3%). It must be considered that the array's spatial resolution is about twice the motion amplitude, which reduces the detector's sensitivity to observe differences in M1S0. This sensitivity could be even lower in uniform dose distributions. However, this finding is consistent with conventional radiotherapy, where small movements are traditionally compensated with an internal tumor volume (ITV) margin.^27^

For array‐based M1S1 and M1S1c comparison, there were no significant differences for 3%/3 mm (< 1%) across all motion ranges. This suggests that kV dose subtraction may not be necessary under the TG‐306 recommended criteria.

For film‐based measurements of the lung case, up to 59.6% and 24.1% more gamma passing points were observed for M1S1 using 2%/2 mm and 1%/1 mm, respectively. Although the AAPM Task Group 76^28^ advises the utilization of motion management techniques for respiratory motion greater than 5 mm in any direction, these findings underscore the importance of employing target motion compensation techniques even for 3 mm motion when high precision is required, especially for SABR treatments.

Limitations and benefits were identified for both setups. The array‐based setup exhibits partial volume effects when targets are comparable in size to the detector's spatial resolution (∼10 mm), but it also allows a quick and simple setup while accounting for kV‐doses. However, array‐based devices can yield artificially high results compared to film.

The film‐based setup, although it may overlook the total delivered dose due to kV‐underresponse and is associated with intrinsic uncertainties from more complex handling, is ideal for PSQA of small targets and periodic quality controls. Its higher spatial resolution and sensitivity make it particularly effective for detailed assessments of Synchrony's performance.

### 4.2 Clinical implementation

Synchrony “simulation,” or delivery feasibility, is a crucial stage during clinical implementation, established with two primary objectives: First, it assesses the system's capability for accurate tracking, especially in lung cases, where the “Lung with Respiratory” tracking method needs verification, or fiducial implantation could be required. Second, it helps to determine the target's motion range, facilitating the selection of jaw‐size for optimal treatment planning efficiency. It is recommended to use a planning scheme with a jaw‐size of 1.0 cm during simulation to achieve the widest tracking capability from the collimation system. However, it is advisable for users to find a balance between treatment speed, dose per fraction, dose uniformity, and OAR sparing. This approach ensures that the simulation plan is not excessively lengthy while providing sufficient duration for respiratory pattern analysis and system evaluation.

For patients with a target motion range equal to or less than 11 mm, a treatment plan was established with a jaw size of 2.5 cm. This configuration ensures that, during treatment, if the motion exceeds the system's correction capacity, the tracking model remains within a minimal percentage of the target outside the jaw range, set at 1%. In cases where the range of movement exceeds 11 mm, planning will employ a 1.0 cm jaw size, allowing for broader MLC‐ and Jaw‐correction and minimizing shortcomings in the tracking model. In situations where the movement range surpasses the system's maximum nominal correction capability (± 20 mm), an increase in the parameter “percentage of the target outside the jaw range” should be considered according to the PTV's expansion margin.

The results for Synchrony validation tests using established harmonic patterns allowed us to evaluate the system's performance and limitations. However, performing PSQA tests with real motion‐traces obtained from Synchrony‐simulation provides a more accurate assessment of the system's ability to track erratic and unexpected movements. This approach could help determine whether margin expansion or even reduction is necessary based on the system's accuracy. The accuracy of the predictive modeling in Synchrony is highly dependent on the stability and repeatability of the target trajectory. For very stable target trajectories, such as harmonic motion traces, the predictive modeling is expected to be highly precise. However, for patient‐specific target trajectories, the modeling will become less accurate and repeatable, potentially leading to lower gamma passing results.

Standardized Synchrony‐thresholds for parameters such as Tracking‐Range, Rigid‐Body, Target‐Offset, Potential‐Diff, and others are not reported, as they are specific to each patient. Additionally, their modification between treatment fractions may be required. This is why validating the system before clinical implementation provides a powerful tool for clinical support, allowing familiarity and experience with handling Synchrony parameters. A clinical protocol for SABR treatments using the “Lung with Respiratory” method was implemented, considering potential outcomes through a review of the system's ability to deliver the prescribed dose, gamma index and criteria, and the dosimetric impact of motion compensation. This process involves the Synchrony‐simulation and the previous analysis. Table 5 provides a tool for treatment selection according to targets characteristics and provides recommendations for dynamic‐PSQA evaluation.

## 5 CONCLUSION

The proposed array‐ and film‐based setups allowed for the successful evaluation of Synchrony operation and performance. Synchrony target tracking and motion compensation exhibited accurate and reliable functioning. The innovative approach of positioning external fiducial markers on the phantom transformed a conventional IMRT detector system into a dynamic evaluation device trackable with Synchrony, particularly with “Fiducial with Respiratory” tracking mode. This methodology can be extended to other devices, especially those allowing a quick and easy setup, preferably with high spatial resolution.

For both array‐ and film‐based setups, similar dosimetric trends were observed. As expected, dose blurring in M1S0 cases emphasizes the need for target motion compensation, particularly for extended beam‐on times or high‐dose treatments. The use of Synchrony (M1S1) showed a statistically noteworthy enhancement in comparison with the M1S0 scenario for all motion ranges. However, the array‐based setup showed an apparently superior gamma passing rate during the “Lung Case” due to the array detector lower spatial resolution compared to film. Despite this, the array‐based configuration was deemed suitable to evaluate most treatments/target‐sizes with Synchrony, providing a fast and straightforward setup. Additionally, array‐based methods allowed for an easier estimation and correction of the kV dose contamination.

Clinically tight gamma‐criteria were employed to better highlight subtle changes in the dose distribution for the proposed dynamic setup and to explore Synchrony performance in detail. This methodology also offered a tool for determining the necessity and extent of target expansion margins. Although the evaluation with the array‐based method shows acceptable M1S1 gamma passing rate results for all motion ranges, film‐based evidence shows a considerable gamma‐passing decrease because of its higher spatial resolution. This implies that the resolution of the array‐based configuration is not advisable for evaluating dose distributions of small targets with stringent criteria (such as 1%/1 mm or 2%/2 mm). However, it is suitable for larger cases and when kV‐dose correction is addressed. Film‐based setup is then recommended for small targets. We advise, though, against using tighter gamma‐criteria with this setup due to the uncertainties related to manipulation, setup, processing, and densitometry, which could lead to an inadequate evaluation of Synchrony PSQA. Findings regarding kV‐dose contamination can be considered negligible for Synchrony's kV energy and the range of kV dose delivered.

The kV dose is an emerging concern according to the upcoming ICRP TG‐116.^29^ We recommend including the Synchrony‐Simulation feature to develop strategies for mitigating its impact. These strategies include selecting appropriate kV protocols according to the treatment and patient's anatomy, presetting the minimum necessary radiographs per rotation, and choosing the most suitable tracking method. Synchrony‐Simulation also provides motion amplitude information to optimize treatment planning parameters, such as jaw‐field size. Although extracting real‐patient motion traces is possible, support from the manufacturer is required. The proposed PCC Table serves as a reference tool for patient and treatment selection, gamma evaluation criteria, and dosimetric considerations in dynamic‐PSQA evaluations. Both the Synchrony‐Simulation and the PCC Table are clinically useful tools that support personalized evaluation and the safe implementation of Synchrony treatments.

Furthermore, it is recommended that new Synchrony users adopt a similar procedure as a guideline for machine characterization during initial system validation and clinical implementation. We advocate for conducting PSQA for the initial patients in each new treatment modality, fiducial or fiducial‐free treatments. An average motion tracking model derived from each case can facilitate periodic evaluations, thus mitigating the need for individual motion trace extraction. New users should also explore strategies to mitigate the overresponse of kV dose for certain array‐detectors; when kV‐protocol characterization has been addressed, the impact of unaccounted kV‐doses during patient treatment can be minimized by using the kV‐protocol with the lowest effective dose.

Although many other considerations were not evaluated in this work such as delivery phase difference between the internal target and external surrogate dependency, LED accuracy, different guidelines such as AAPM TG‐306, TG‐147, and TG‐135 describe testing that can be included during system evaluation. As complementary work, it is proposed to develop a gamma index evaluation dependency on the target size and dose per fraction. This could extend the scope of what is proposed in the PCC table to classify and estimate the possible results of gamma passing rate according to any dose criteria for any possible treatment with Synchrony.

## AUTHOR CONTRIBUTIONS

Christian David Trujillo‐Bastidas: Conceived and designed the experiments presented in the accompanying manuscript, acquired the data included in the manuscript, and conducted the analysis and interpretation of the data. Additionally, drafted the initial manuscript and adjusted the manuscript to the publication format of the journal. Michael James Taylor: Made substantial contributions to the collection and analysis of data, analyzed, and interpreted the data presented in this manuscript. Provided critical feedback on the accompanying manuscript and participated in the revision and editing process. Finally, gave final approval for the publication of the accompanying manuscript. Gloria María Díaz‐Londoño: Provided guidance and oversight throughout the project. Offered critical feedback on the accompanying manuscript and actively participated in the revision and editing process. Ultimately, granted final approval for the publication of the accompanying manuscript.

## CONFLICT OF INTEREST STATEMENT

Michael Taylor is employed by Accuray Inc. The remaining authors have no conflicts of interest to disclose.
